# Doxorubicin-induced ovarian toxicity

**DOI:** 10.1186/1477-7827-8-20

**Published:** 2010-03-04

**Authors:** Irit Ben-Aharon, Hadas Bar-Joseph, Galia Tzarfaty, Lital Kuchinsky, Shulamith Rizel, Salomon M Stemmer, Ruth Shalgi

**Affiliations:** 1 Institute of Oncology, Davidoff Center, Rabin Medical Center, Beilinson Campus, Petah-Tiqva, Israel; 2 Department of Cell and Developmental Biology, Sackler Faculty of Medicine, Tel Aviv University, Israel; 3 Department of Radiology, Sheba Medical Center, Tel Hashomer, Israel

## Abstract

**Background:**

Young cancer patients may occasionally face infertility and premature gonadal failure. Apart from its direct effect on follicles and oocytes, chemotherapy may induce ovarian toxicity via an impact on the entire ovary. The role of doxorubicin in potential ovarian failure remains obscure. Our intention was to elucidate doxorubicin-related toxicity within ovaries.

**Methods:**

Female mice were injected intraperitoneally with 7.5 or 10 mg/kg doxorubicin and their ovaries were visualized in vivo by high resolution MRI, one day and one month following treatment. Ovaries of other treated mice were excised and weighed at the same post-treatment intervals. Ovarian histological sections were stained for TUNEL or active caspase-3 and follicles were counted and categorized. Ovulation rates were evaluated in superovulated female mice treated with doxorubicin.

**Results:**

A single injection of doxorubicin resulted in a major reduction in both ovarian size and weight that lasted even one month post treatment. A dramatic reduction in ovulation rate was observed one week after treatment, followed by a partial recovery at one month. Histological examination revealed positive staining of TUNEL and active caspase-3. We observed a significant reduction in the population of secondary and primordial follicles one month following treatment.

**Conclusions:**

Our results may imply a mechanism of chemotherapy-induced ovarian toxicity, manifested by reduced ovulation and accompanied by a reduction in ovarian size, caused probably by an acute insult to the ovary.

## Background

Recent advances in cancer therapy have improved the long-term survival of young cancer patients who then may face iatrogenic infertility and premature gonadal failure [1]. Although the need for tumor eradication in these patients is clear, the long-term effects of chemotherapy, observed on non-target tissues such as ovaries, are substantial and raise concern and potential health risks in women surviving diseases as breast cancer, Hodgkin's disease and leukemia. The assessment of chemotherapy-induced gonadal toxicity is based mainly on indirect parameters as menstrual history, incidence of amenorrhea, hormones levels and surveys of subsequent parity [1-4]. Former studies had demonstrated apoptotic changes in the pregranulosa cells that result in follicular damage [5,6], injury to blood vessels and focal ovarian cortical fibrosis [7] following cyclophosphamide administration. The outcome of ovarian damage due to chemotherapy depends on the size of the follicular reserve, which is age-related [1]. According to these studies, chemotherapy may affect the entire ovary aside from the direct effect it exerts on the follicles and oocytes. Alkylating agents play a role in inducing premature ovarian failure [5,8,9]. Though it has been demonstrated that doxorubicin, an anthracycline agent, arouses apoptosis in oocytes, the role of anthracyclines in inducing ovarian failure remains obscure [6,10,11]. The risk of developing amenorrhea following treatment with doxorubicin-containing protocols ranges from 20% to 80%, depending on the woman's age (high incidence at the age of 40 and older; moderate incidence at the age of 30-39; 1,4). The pattern of chemotherapy-induced ovarian injury may consist of endovascular damage since signs of fibrosis in the cortical stroma and changes in the capillaries were observed [7,12]. Analogous pattern was presented also in biopsies retrieved from patients who had been treated with doxorubicin-based chemotherapeutic regimens [13].

In the current study we have established a platform of *in vivo *imaging for investigating the short and long term effects of doxorubicin on the ovaries. We have characterized the pattern of mice ovarian toxicity, induced following an *in vivo *administration of doxorubicin. First, we characterized the mode of tissue impairment, by assessing the pattern of apoptosis within the affected ovary. We then followed the modifications in ovarian function, demonstrated by an altered ovulation rate. A sharp decline in ovary size was observed by *in vivo *imaging, verified by a corresponding decline in ovarian weight. Our results may indicate a mechanism of chemotherapy-induced ovarian toxicity, manifested by reduced ovulation and accompanied by a reduction in ovarian size due to possible acute ovarian insult.

## Methods

ICR female mice (7-8 weeks old; Harlan Laboratories, Jerusalem, Israel) were housed in air conditioned, light controlled animal facilities of the Sackler faculty of Medicine in Tel-Aviv University. Animal care was in accordance with institutional guidelines and was approved by the local authorities. Doxorubicin (Teva; Petach-Tikvah, Israel) and saline were injected at a volume of 100 μl/10 gr body weight.

### Ovary weight

Four weeks old immature ICR female mice were injected intraperitoneally (IP) with either 7.5 mg/kg doxorubicin in saline or with saline alone (control) and their ovaries were excised and weighed one week or one month later. The ovaries of 4-weeks old females are comprised mostly of follicles, without corpora lutea.

### Ovulation rate

We studied the effect of a single injection of doxorubicin (10 mg/kg) on ovulation rate in 7-8 weeks old ICR female mice. Control mice were injected IP with saline. Ovulation was induced by injecting the mice with 5 IU human chorionic gonadotropin (hCG; Sigma, St Louis, MO, USA) 48 hours after administration of 10 IU pregnant mare serum gonadotropin (PMSG; Syncro-part, Sanofi, Paris, France). The mice were sacrificed 16-17 hours after hCG administration at a time coinciding with either 48 hours, one week or one month after the doxorubicin or saline injection. Ovulated oocytes were isolated from the oviductal ampullae into Toyoda HEPES (TH) medium [14] supplemented with 0.4% BSA (fraction V, Sigma) and counted.

### High resolution magnetic resonance imaging (MRI) for measuring ovarian size

Female mice, 7-8 weeks old were injected IP with 7.5 mg/kg doxorubicin (n = 9) or with saline (n = 9) and subjected to MRI imaging of their ovaries. In order to avoid the inter-individual variability, the same mice were anesthetized before the injection (baseline) as well as 1 day and 1 month after the injection, for repeated imaging of their ovaries. Changes in ovary size and in amount of fluids in the peri-ovarian bursal sac were recorded.

Mice were anesthetized with isoflurane, (5% in oxygen for induction, 1-3% for maintenance) at a rate of 1 liter/min. Once anesthetized, the mice were installed in a head-holder to assure a fixed positioning inside the probe throughout the entire measurement period. Respiration rate was monitored (60-80 breaths/min) throughout the experimental period.

MRI experiments were performed using the 7 Tesla BioSpec Magnet 70/30 USR system (Bruker, Germany) equipped with a gradient coil system capable of producing pulse gradient of up to 40 gauss/cm in each of the three directions. The MRI protocol included coronal T2-weighted images that were acquired using the RARE sequence, 256 × 128 matrix (interpolated to 256 × 256), RARE factor of 8, and four averages, corresponding to an image acquisition time of 3 min 44 sec. Twenty four continuous slices of 0.85 mm thickness were acquired with a field of view (FOV) of 6 cm × 6 cm.

The ovary volume was calculated from the T2-weighted MR images using the Medical Image Analysis (MIA version 2.4), MATLAB^® ^image processing toolbox.

### Apoptosis within ovaries

Four weeks old mice were excised 12 or 24 hours after administration of 10 mg/kg doxorubicin or saline, fixed in 4% paraformaldehyde, paraffinized and sectioned. Immunohistochemistry was performed on deparaffinized, paraformaldehyde-fixed sections of the excised mice ovaries: TUNEL assay Roche-Applied-Science) for detection of DNA fragmentation and caspase-3 assay for detection of apoptosis. Caspase-3 assay was performed using an anti-caspase 3 antibody (cleaved caspase-3; Cell Signaling Technology, MA, USA) according to Asheri-Padan et al. [15]. Labeled sections were visualized and photographed by a confocal laser-scanning microscope (Leica TCS SP5; Mannheim, Germany), equipped with an argon-ion laser (458 nm, 476 nm, 488 nm, 496 nm, 514 nm lines), a diode-pumped solid state laser (516 nm line) and an UV diode laser. Water-immersion lenses (20× NA/0.7 and 63× NA/1.2) were used for all imaging. The sections were scanned using the confocal laser-scanning microscopy program (Leica microsystem LAS AF).

### Classification of follicles

Four weeks old mice were injected IP with 7.5 mg/kg doxorubicin, their ovaries excised one month later, fixed in Bouin's solution, embedded in paraffin, sectioned (6 μm) and mounted on Superfrost/Plus slides (Daigger and Co., Wheeling, IL, USA). The Bouin-fixed ovarian sections were stained with Hematoxylin-Eosin to detect doxorubicin-induced variations in structure and number of follicles. All ovarian follicles of every fifth consecutive histological section were classified according to type and counted. The numbers were multiplied by 5 to reach a value representative of the total number of follicles within an ovary as formerly described [16].

### Statistical analysis

Each experiment was repeated at least three times. SPSS 10.0 software was used for statistical analysis. Two-way analysis of variance (ANOVA) test was employed for assessing the degree of correlation between counted follicles of ovaries from treated and control groups. ANOVA with repeated measures was used for assessing differences in ovulation rate, ovarian weight and size (as measured with MRI), between treated and control mice. Results were considered statistically significant at P < 0.05.

## Results

### The effect of in vivo doxorubicin administration on ovarian weight

**Figure 1:**
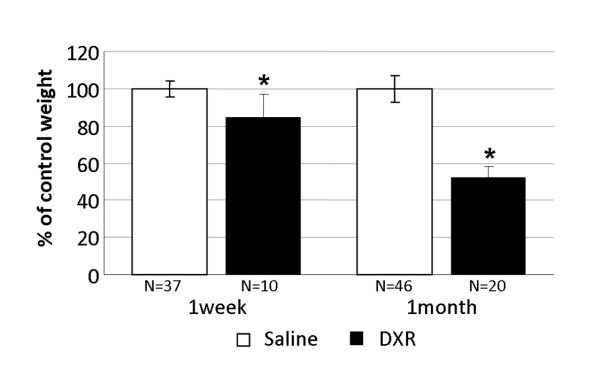
**Changes in ovarian weight induced by doxorubicin**. Ovaries of female mice injected with doxorubicin were excised, weighed one week or one month after injection and compared with those of saline-injected control mice. Bars represent weight (mean ± SEM) of ovaries, (*)-significantly different from control value (P < 0.05); N = number of ovaries.

The weights of ovaries excised from female mice either one week or one month after injection of doxorubicin were compared to weights of ovaries excised from saline-injected control mice at the same post-injection time intervals. A time-dependant relative decrease in ovarian weights was detected: from 85% of control value at one week after treatment to 52% of control value one month post treatment (P < 0.05; Fig. 1).

### The effect of in vivo doxorubicin administration on ovulation rate

**Figure 2:**
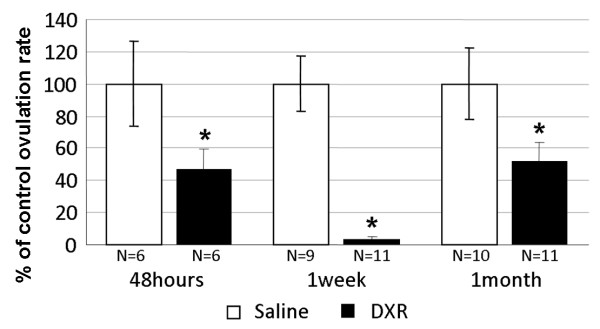
**Ovulation rate following doxorubicin treatment**. Female mice were injected with doxorubicin or with saline. Ovulation was induced by injecting the mice with 5 IU hCG 48 hours after administration of 10 IU of PMSG. The mice were sacrificed 16-17 hours after hCG administration at a time coinciding with either 48 hours, one week or one month after the doxorubicin or saline injections. The number of ovulated oocytes was recorded. Bars represent percent of ovulated control oocytes (mean ± SEM), (*)-significantly different from control value (P < 0.05); N = number of mice.

Doxorubicin-treated female mice were injected with PMSG and hCG to induce superovulation. Ovulation rate was decreased from about half of control value at 48 hours to 4% at 1 week and recovered back to 52% one month after treatment (Fig 2).

### In vivo imaging of ovaries after doxorubicin treatment

**Figure 3:**
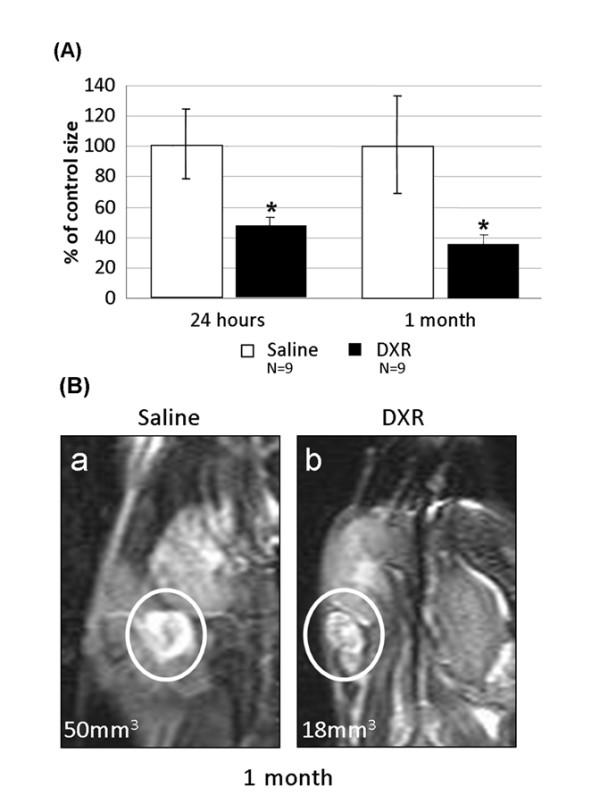
**The effect of doxorubicin on the size of ovaries**. The ovaries of anaesthetized mice injected with doxorubicin or with saline were imaged with a high-resolution coronal T2-weighted MRI 24 hours and one month post injection. (A) A relative decline in ovaries size was observed already 24 hours post treatment and persisted also one month post treatment. Bars represent percent of control (mean ± SEM), (*)-significantly different from control value (P < 0.05); N = number of mice. (B) Representative images of ovaries from saline-injected (a) and doxorubicin-injected (b) mice at one month post treatment.

The use of high-resolution MRI enabled live imaging of mice ovaries with repeated monitoring of the same individuals thus minimizing variability. Post-treatment changes in the size of mice ovaries at various time intervals were tracked by this imaging platform. Since ovaries of rodents are enclosed in a periovarian sac, the bursa, we measured dimensions of both bursa and ovary in an attempt to follow possible variations in size occurring as a result of doxorubicin treatment. A continuous reduction in the size of ovaries over time (Fig 3A), with fluctuations in bursal dimensions were observed., A peri-ovarian edema, manifested by a shrunken ovary and a swollen bursa were detected during the acute phase, 24 hours following doxorubicin treatment. The trend of decline persisted also one month after treatment (Fig 3B), consistent with the relative reduction in ovary weight but inconsistent with the partial recovery in ovulation rate reported in a former section.

### Induction of follicular apoptosis by doxorubicin

**Figure 4:**
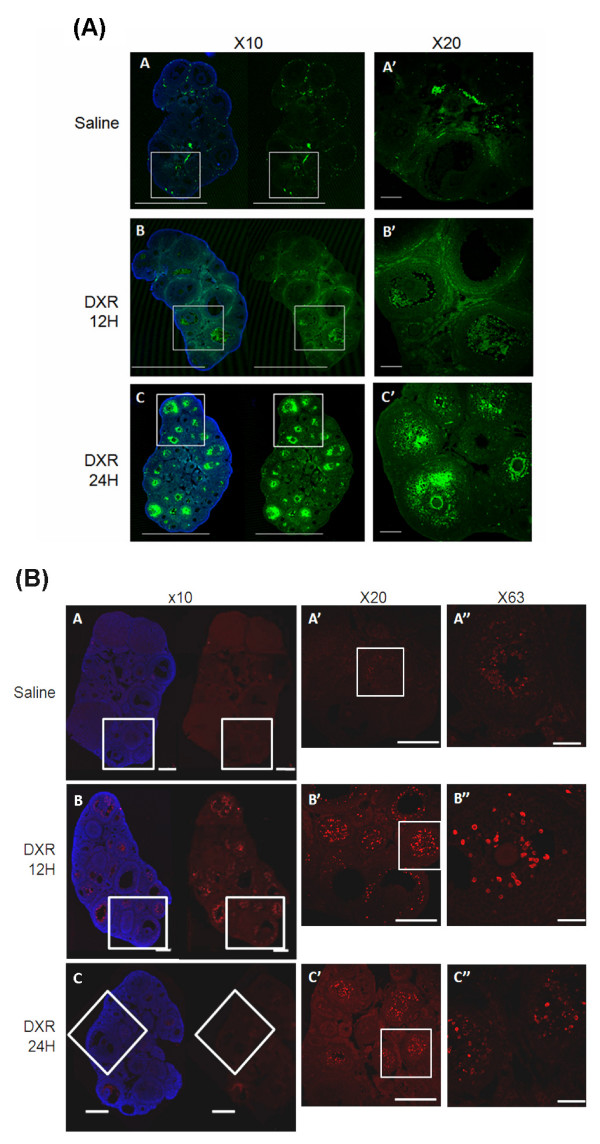
**Doxorubicin induced ovarian apoptosis**. Confocal micrographs of ovaries excised from doxorubicin or saline-injected mice, 12 or 24 hours after treatment. Ovaries of saline-treated mice (A, A'A"). Ovaries of doxorubicin-treated mice 12 hours (B, B'B") or 24 hours (C, C'C") after treatment. Magnification: ×10 (A-C), ×20 (A'-C'), ×63 (A"-C"). (A) TUNNEL staining (green), (B) caspase-3 staining (red). Blue staining represents DNA labeling by Heochst 32242.

Ovaries of female mice injected with doxorubicin were excised, fixed and sectioned at various time points after exposure to the agent. Occurrence of DNA fragmentation and apoptosis in ovarian follicles were assessed in parafrmaldehyde-fixed histological sections by TUNEL and active caspase-3 assays, respectively, 12 or 24 hours post treatment. Fluorescence microscopy revealed positive TUNEL reaction in secondary and pre-antral follicles, but most prominently in antral follicles. The reaction signal was weakly evident 12 hours after doxorubicin treatment, mostly in the granulosa compartment, enhanced and shifted towards the oocyte 24 hours post treatment. The primordial follicles did not display staining in this assay while primary follicles were faintly stained (Fig 4A).

Active caspase-3 was clearly manifested in secondary follicles, pre-antral and antral follicles 12 hours following doxorubicin treatment, corresponding with the location of TUNEL staining, although caspase 3 staining was evident earlier than the TUNEL reaction (Fig 4B).

### The effect of doxorubicin on the follicular population within the ovary

**Figure 5:**
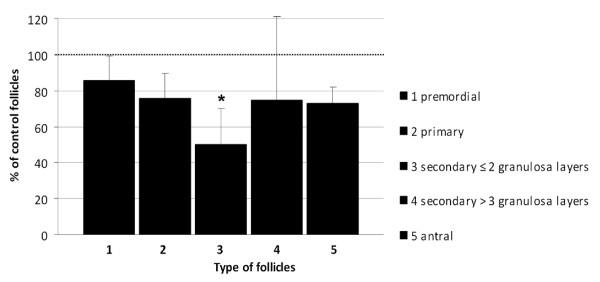
**Depletion of ovarian follicles reservoir by doxorubicin**. The number of ovarian follicles (mean ± SEM) at various developmental stages, one month after injection of doxorubicin or saline. Bars represent mean ± SEM, (*)-significantly different from control value (P < 0.05).

Bouin-fixed ovaries, one month post treatment, were sectioned and stained by Hematoxylin-Eosin. The ovarian follicles were counted and categorized according to their developmental stage. The population size of each type of ovarian follicles was compared between ovaries from doxorubicin and saline injected mice. The size of secondary follicles population, one month after doxorubicin administration, was significantly reduced compared to the control group (p = 0.03; Fig 5). The population of primary, primordial and antral follicles also decreased in size one month after doxorubicin treatment, thugh not statistically significant (p = 0.06 and 0.08, respectively).

Furthermore, the ovarian cortex of doxorubicin-treated mice appeared more vascular, with thickening and hyalinization of the vessels wall. The vascular changes were evident in sections of ovaries excised one month following doxorubicin administration (data not shown)

## Discussion

In this study we explored the possible patterns of apoptotic processes initiated within the mouse ovary following exposure to the widely used chemotherapeutic agent, doxorubicin. The dose employed was equipotent to that used in humans, specifically in breast cancer patients. The observation, depicted by *in vivo *MRI imaging, of a decrease in ovarian size occurring after doxorubicin administration was further validated by a similar effect on ovarian weight, though the mean weight of ovaries excised from doxorubicin-treated mice at various post-treatment time points might also reflect inter-mice variability. This variability was minimized by repeatedly imaging with MRI the same treated individuals at the same post-treatment intervals. An acute effect, observed by MRI within the first 24 hours post treatment, indicated a marked peri-ovarian edema that may represent actual ovary shrinkage, caused by a shift of fluids from the ovarian tissue to the surrounding bursal sac (or peritoneal space in human). This phenomenon may resemble the radiological phenomenon referred to as 'Page kidney', where an external compression of the kidney causes renal hypoperfusion and ischemia [17]. Some studies suggested that this pathogenesis involves "parenchymal scarring" of the kidney [18]. Though we suggest that our observation of doxorubicin-induced impaired ovarian size and weight may involve an analogous mechanism of an acute ovarian insult due to ischemia (which may be followed by parenchymal fibrosis), the matter should be further explored. The effect on ovary size persisted even one month post treatment. The "one month" time point was chosen since it correlates to 6-7 menstrual cycles (6-7 months), the estimated number of cycles required for human follicular growth and maturation [19]. Despite the increased impairment in ovary size over time, the decrease in ovulation rate following exposure to doxorubicin was partially reversed. However, as formerly reported, the reproductive performance of cyclophosphamide treated female mice, expressed by ovulation; mating and pregnancy rate was not compromised compared to control mice, although most of the population of primordial follicles had been destroyed. Nonetheless, a considerable reduction in the number of pregnancy sacs was observed in females mated one week after cyclophosphamide treatment, which may suggest that mature follicles are more sensitive to chemotherapy-induced damage [9]. In our study doxorubicin injected mice presented compromised ovulation rates and hence also reduced blastocysts count in mated mice of this group. Furthermore, we observed many atretic antral follicles within the ovaries at 24 hours and one month after doxorubicin administration, which correlated with the significant reduction in ovulation rate. Nevertheless, oocytes ovulated by doxorubicin-treated mice revealed neither morphological nor chromosomal changes compared with oocytes ovulated by control mice (data not shown). Hyperstimulating the ovaries with gondaotropins recruits gonadotropins-responsive follicles (i.e., secondary follicles). The partial recovery of ovulation rate depicted one month after doxorubicin administration correlates with the physiological time-span required for the mouse primary follicles to mature.

In an attempt to characterize the apoptotic effect of doxorubicin on the ovary, we were able to detect apoptosis in histological sections of mice ovaries by depicting caspase-3 activity, as soon as 12 hours following exposure to the agent. TUNEL staining was noticeable only when the apoptotic end-products accumulated, 12-24 hours following doxorubicin administration. Further histological changes, induced by doxorubicin in the stromal portion of the ovary were observed. It had formerly been documented that ovarian histological biopsies from women who had received various chemotherapeutic regimens, exhibited altered ovarian stromal function as reflected by lower *in vitro *production of estradiol, regardless the presence of an alkylating agent in the chemotherapeutic regimen and irrespective of the magnitude of germ cell damage [20]. We observed fibrosis of the cortical vessels of ovaries of doxorubicin treated but not of control mice. It has been previously shown that doxorubicin at submicromolar concentrations induces in endothelial cells apoptosis that is mediated presumably by formation of oxidative free radicals and causes impaired endothelial function [21-23]. Furthermore, cortical fibrosis had already been described in histological sections of ovarian biopsies taken from women receiving chemotherapy [7]. The loss of premature follicles, as well as the perivascular and parenchymal changes in ovaries of mice treated with doxorubicin *in vivo*, correlate with the histological modifications demonstrated in human ovaries following administration of other chemotherapeutic agents such as cyclophsphamide and cisplatin [5]. These studies hypothesized a combined mechanism of neovascularization and ovarian tissue scarring with a direct toxic effect on the primordial follicles. Mice primordial follicles are destroyed following administration of the chemotherapeutic drug cyclophosphamide [9]. We had presented quantitative evidence that *in vivo *doxorubicin treatment results in a significant loss of follicular reserve within mice ovaries, mostly secondary follicles, and a nearly statistical significant trend of decline in the number of primordial and primary follicles. Populations of all follicles were damaged, significantly the secondary follicles. The primordial follicles presented a trend consistent with published data of other chemotherapies. The pronounced depletion of the follicular population was observed one month following exposure to the agent (equivalent to 6-7 months in human). As mentioned, there is former evidence in the literature for loss of primordial follicles in rodents treated with alkylating agents [9] as well as in women treated with alkylating agents-based protocols [13,20]. According to previous studies and daily practice, the ovarian reproductive potential of chemotherapy survivors is assessed mainly by documenting menstrual activity or specific hormonal markers in serum. Since menstruation does not accurately reflect the fertility state [22,24,25], assessing the population of primordial follicles in the ovary may serve as a better indicator.

The follicular reserve within the ovaries consists mainly of quiescent primordial follicles developed during fetal life. A tremendous number of primordial follicles will be annihilated, before or shortly after birth and throughout postnatal life, by a physiological programmed cell death process (i.e., apoptosis(; [26]. Caspases, cysteine proteases synthesized as pro-enzymes and activated by autocatalytic cleavage or by other proteases, are considered as key effectors during the apoptotic cascade. It is well established that caspase-3 plays a pivotal role in the execution of apoptosis and is predominantly localized in granulosa cells of atretic follicles. It has been suggested that caspase-3 might be involved in the physiological follicular atresia within ovaries [27,28]. This physiological cellular machinery may predispose the follicles to apoptosis induced by exogenous signals, such as chemotherapeutic agents. This trait may correlate with the higher rate of premature ovarian failure observed in women following chemotherapy treatment [1]. Hence, due to the decrease in primordial follicles pool size throughout physiological aging, the rate of ovarian failure observed in older women treated with chemotherapy is higher than in younger women [1,29].

Since the effect of doxorubicin was evident both microscopically at both cell and organ levels, we suggest a whole-organ effect of the agent. *In vivo *imaging of the ovaries served as a tool for tracking down the whole organ pattern of the gonadotoxic effect of doxorubicin and may be used as a platform for future investigation of agents needed to shield the ovaries from chemotherapy peril.

## Competing interests

The authors declare that they have no competing interests.

## Authors' contributions

HB carried out the biochemical studies and confocal imaging, performed the statistical analysis and drafted the manuscript. IB participated in the design of the study and drafted the manuscript. RS conceived of the study, and participated in its design and coordination and helped to draft the manuscript and supervised the study. SS and RS participated in the design of the study. MT carried out the caspase assays. All authors read and approved the final manuscript.
